# Spinal Shox2 interneuron interconnectivity related to function and development

**DOI:** 10.7554/eLife.42519

**Published:** 2018-12-31

**Authors:** Ngoc T Ha, Kimberly J Dougherty

**Affiliations:** Department of Neurobiology and AnatomyDrexel University College of MedicinePhiladelphiaUnited States; Emory UniversityUnited States; Emory UniversityUnited States

**Keywords:** spinal cord, rhythm generation, locomotion, synaptic connectivity, gap junctions, Mouse

## Abstract

Neuronal networks generating hindlimb locomotion are located in the spinal cord. The mechanisms underlying spinal rhythmogenesis are unknown but network activity and interconnectivity of excitatory interneurons likely play prominent roles. Here, we investigate interconnectivity within the Shox2 interneuron population, a subset of which has been suggested to be involved in locomotor rhythm generation, using paired recordings in isolated spinal cords or slices from transgenic mice. Sparse unidirectional connections consistent with chemical synaptic transmission and prominent bidirectional connections mediated by electrical synapses were present within distinct subsets of Shox2 interneurons. Moreover, bidirectional electrical connections were preferentially found between functionally-related Shox2 interneurons. Though prevalent in neonatal mice, electrical coupling began to decline in incidence and strength in mice ~ 3 weeks of age. Overall, our data suggest that gap junctional coupling promotes synchronization of Shox2 interneurons, and may be implicated in locomotor rhythmicity in developing mice.

## Introduction

Central pattern generators (CPGs) are neural networks that can generate and control the rhythm and pattern of muscle activation, even in the absence of supraspinal input and sensory feedback. Such networks underlie many repetitive motor behaviors in vertebrates including chewing, breathing, and walking. Among these, the CPG controlling hindlimb locomotion is intrinsic to the spinal cord (Grillner, 2006; Kiehn, 2016), and consists of rhythm-generating neurons and neurons participating in pattern formation. Although several constituent classes of locomotor CPG neurons have been identified based on transcription factor expression, most function as patterning neurons involved in left-right or flexor-extensor coordination (Garcia-Campmany et al., 2010; McLean and Dougherty, 2015; Rybak et al., 2015; Kiehn, 2016; Ziskind-Conhaim and Hochman, 2017). Rhythm-generating neurons have been more elusive as no single genetically identifiable population has been shown to be solely responsible for the rhythm.

Rhythm-generating neurons are excitatory, ipsilaterally-projecting, and mutually connected neurons (Buchanan and Grillner, 1987; Bracci et al., 1996; Kjaerulff and Kiehn, 1996; Kjaerulff and Kiehn, 1997; Li et al., 2006; Li et al., 2009; Hägglund et al., 2010; Hägglund et al., 2013). Two glutamatergic, ipsilaterally-projecting interneuronal populations have been identified as containing candidate rhythm-generating neurons. These neurons are identified by developmental expression of either the transcription factor Shox2 or Hb9 (Hinckley et al., 2005; Wilson et al., 2005; Dougherty et al., 2013; Caldeira et al., 2017). Both Shox2 and Hb9 interneurons (INs) express many of the hallmark features of rhythm-generating neurons (Brownstone and Wilson, 2008; Kiehn, 2016). Shox2 INs are located in the intermediate zone throughout the rostral-caudal extent of the spinal cord and Hb9 INs are in the ventromedial region, restricted to segments T13-L3 (Hinckley et al., 2005; Wilson et al., 2005). Shox2 INs can be further subdivided into two groups based on the expression of Chx10, the marker of V2a INs. Genetic manipulation experiments suggest that Shox2^+^ non-V2a (Chx10^-^) INs are part of the locomotor rhythm generator, while Shox2^+^ V2a (Chx10^+^) INs belong to the locomotor circuitry but are not directly involved in rhythm generation (Dougherty et al., 2013).

A key feature of rhythm-generating neurons is their ability to convert descending signals from hindbrain into a rhythmic output, which is transmitted to the circuit. The precise mechanisms by which this occurs in mammalian locomotor CPGs have been poorly described due to the difficulty in targeting specific populations of rhythm-generating neurons. Regardless of mechanism (Brownstone and Wilson, 2008; Brocard et al., 2010; Harris-Warrick, 2010; Kiehn, 2016), connections between these neurons would be necessary to generate a synchronized rhythmic population activity. Therefore, neuronal connectivity is likely to play a significant role in locomotor rhythm generation.

In vertebrates, the majority of neuronal connectivity is mediated by chemical synapses but electrical synapses are also prevalent in neonatal rodent spinal cord (Chang et al., 1999; Kiehn and Tresch, 2002; Hinckley and Ziskind-Conhaim, 2006; Bautista et al., 2012). Functional evidence of gap junctional coupling has been extensively reported in neonatal rodent spinal cord and brainstem preparations with a decline as the animal matures (Chang et al., 1999; Kiehn and Tresch, 2002; Mentis et al., 2002; Hinckley and Ziskind-Conhaim, 2006; Lee et al., 2005; Bautista et al., 2012); however connexin proteins can be detected in the adult spinal cord (Rash et al., 1996; Chang et al., 1999; Nagy et al., 2004; Rash et al., 2000; Personius et al., 2007; Marina et al., 2008; Bautista et al., 2012). Electrical transmission via gap junctions contributes to rhythmic oscillations and neuronal synchrony in many CPGs (Marder and Calabrese, 1996; Rekling et al., 2000; Brownstone and Wilson, 2008). Gap junctions can enhance synchronization and affect frequency of rhythmic activity (Bou-Flores and Berger, 2001), suggesting an involvement in rhythm. Further, blocking gap junctions eliminates most drug evoked locomotion (Tresch and Kiehn, 2000). This may be due to a desynchronization of oscillating motor neurons (Kiehn et al., 2000) and/or a loss of coupling between rhythm-generating interneurons (Hinckley and Ziskind-Conhaim, 2006).

Here, we investigate the connectivity properties of Shox2 interneurons (INs), a population shown to participate in locomotor rhythm generation. Dual whole-cell patch clamp recordings were performed to determine the degree of local connectivity among Shox2 neurons. Recordings were initially performed in dorsal horn-removed preparations and slices from neonatal mice (P0-5) as this is when fictive locomotion is readily elicited in vitro and where function of transcription factor-defined populations was first described. We found two types of interconnections between Shox2 INs: unidirectional connections consistent with chemical synaptic transmission and bidirectional connections mediated by electrical transmission. In more mature preparations, electrical connections between Shox2 INs began to decline around the third postnatal week and could not be detected in adult. Electrical coupling between Shox2 INs is preferential within identified subpopulations of Shox2 INs; therefore allowing for the synchronization of functional populations, particularly in young mice.

## Results

### Unidirectional connections between spinal Shox2 INs are sparse

One feature common to populations of rhythm-generating neurons is mutual excitatory connections between them, which are thought to play a role in rhythmogenesis (Rekling et al., 2000; Li et al., 2006). Therefore, we sought to investigate the interconnectivity between Shox2 INs. We performed whole cell paired recordings from identified Shox2 INs in cords isolated from Shox2::Cre; Rosa26-lsl-tdTomato mice at P0-5. Proof of principle connectivity has previously been shown for a small number of Shox2 IN pairs (Dougherty et al., 2013); however, here, we investigated local Shox2 interconnectivity in detail. Shox2 INs in close proximity were visually identified and targeted based on fluorescence. Initial recordings were performed in the lumbar region of the dorsal horn-removed preparation (84 pairs), as previously (Dougherty et al., 2013); however, the majority of the recordings were done in transverse spinal slices from lumbar cord (155 pairs). Recording from neurons in the slice preparation enables more direct comparison with recordings from more mature animals, which must be performed in spinal slices for visualization, oxygen penetration, and viability. Shox2 INs are primarily short-projecting, so local Shox2 IN connectivity is expected to be largely preserved in the slice preparation (Dougherty et al., 2013).

**Figure 1.**
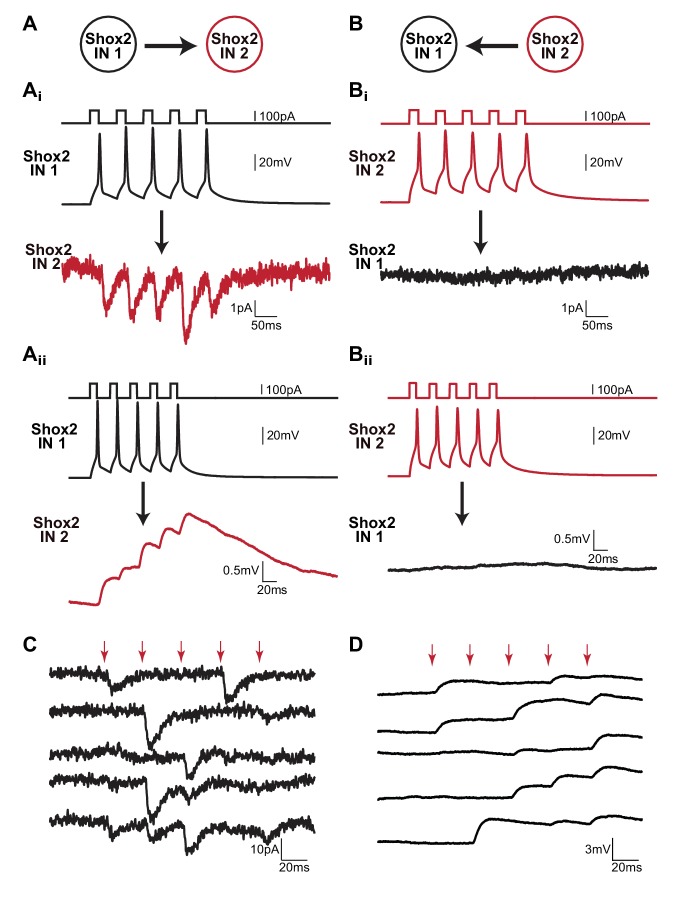
Unidirectional connections are present between Shox2 INs in neonatal mouse spinal cords. (**A** and **B**) Examples of recordings from a pair of Shox2 INs that are unidirectionally connected. (**Ai** and **Bi**) Cartoon of stimulation and recording direction for 2 Shox2 INs. (**Ai** and **ii**) Current was injected in order to evoke five action potentials in Shox2 IN 1 (black). EPSCs (**Ai**) or EPSPs (**Aii**) were evident in Shox2 IN 2 (red) when recorded in voltage clamp or current clamp mode, respectively. Black arrows indicate direction of connectivity tested. (**Bi** and **Bii**) When the protocol was reversed and action potentials were evoked by current injections into Shox2 IN 2 (red), there was a lack of response in Shox2 IN 1 (black). All data shown in (**A** and **B**) were the average of 50 trials. (**C** and **D**) Examples of individual trials from two unidirectionally connected pairs demonstrating that postsynaptic responses did not occur for each presynaptic action potential. The voltage clamp recordings showing EPSCs in (**C**) and the current clamp recordings showing EPSPs in (**D**) are from Shox2 IN 2 in (**A** and **B**). In both (**C** and **D**), red arrows indicate action potential peaks in the presynaptic Shox2 IN.

Connectivity was tested by injecting current to evoke five action potentials in ‘Shox2 IN 1’ and averaging the response of ‘Shox2 IN 2’ in 50 trials (Figure 1A). The protocol was then reversed to measure the response of ‘Shox2 IN 1’ to action potentials evoked in ‘Shox2 IN 2’ (Figure 1B). Consistent with previous findings (Dougherty et al., 2013), a small number of Shox2 IN pairs were connected unidirectionally, such that action potentials evoked by current injections in one Shox2 IN resulted in EPSCs and EPSPs in the other Shox2 IN (Figure 1A_i_ and A_ii_) but not when the protocol was reversed (Figure 1B_i_ and B_ii_). Unidirectional connections between Shox2 INs were sparse (n = 4 of 239 pairs, two in slices, two in dorsal horn removed). This is likely to be an underestimate due to the proximity of the neurons selected for recordings and due to axon and dendritic loss in slicing.

Although the connectivity was evident in the average of 50 sweeps, all unidirectional pairs showed high rates of synaptic failures (Figure 1C and D). This was obvious upon inspection of individual sweeps where postsynaptic responses were not always generated in response to each of the five presynaptic action potentials. In fact, in one postsynaptic neuron, evoked responses were seen in less than 10% of the sweeps. Therefore, for subsequent analysis only sweeps containing a postsynaptic response to the first presynaptic action potential were averaged. At a holding potential of −60 mV, the mean latency between the peak of the first presynaptic spikes and the peak of the first EPSC was 5.0 ± 2.4 ms (n = 3). The mean peak amplitude of the corresponding EPSC was −10.6 ± 3.9 pA. The delay between the peak of the first presynaptic spikes and the onset of the first EPSP was on average 2.0 ± 2.1 ms (n = 3). The mean peak amplitude of the corresponding EPSP was 1.4 ± 0.6 mV. The latency and unidirectional nature of these pairs was consistent with chemical synaptic transmission.

Synaptic failures in response to stimulation of single cells have been observed at other CNS synapses (Bolshakov and Siegelbaum, 1995; Stevens and Wang, 1995; Rekling et al., 2000). It is possible that these chemical connections are not monosynaptic. However, regardless of whether the connections are monosynaptic or disynaptic, it suggests that Shox2 INs may be conditionally recurrently connected. In order to determine if failures were due to immature silent synapses (Kerchner and Nicoll, 2008) or low vesicle release probabilities, subsets of non-connected pairs were tested at positive holding potentials (Liao et al., 1995; Li and Zhuo, 1998; Baba et al., 2000; Yasaka et al., 2009), in serotonin (Li and Zhuo, 1998), or increased extracellular Ca^2+^ (i.e. Chuhma and Ohmori, 1998; Moore et al., 2015). When the postsynaptic Shox2 IN was switched into voltage clamp mode and depolarized to +40 mV to remove the Mg^2+^ block of the NMDA receptors, no additional connections were revealed (n = 0 of 22 pairs, data not shown). Connections were not more likely in 10 µM serotonin (5-HT, n = 0 of 15 pairs) or a high Ca^2+^ (5 mM) solution (n = 0 of 4 pairs), data not shown. Taken together, unidirectional connections were evident in a small population of Shox2 INs, and properties of these connections are consistent with being mediated by chemical transmission.

### Bidirectional connections are present in a large proportion of Shox2 IN pairs

**Figure 2.**
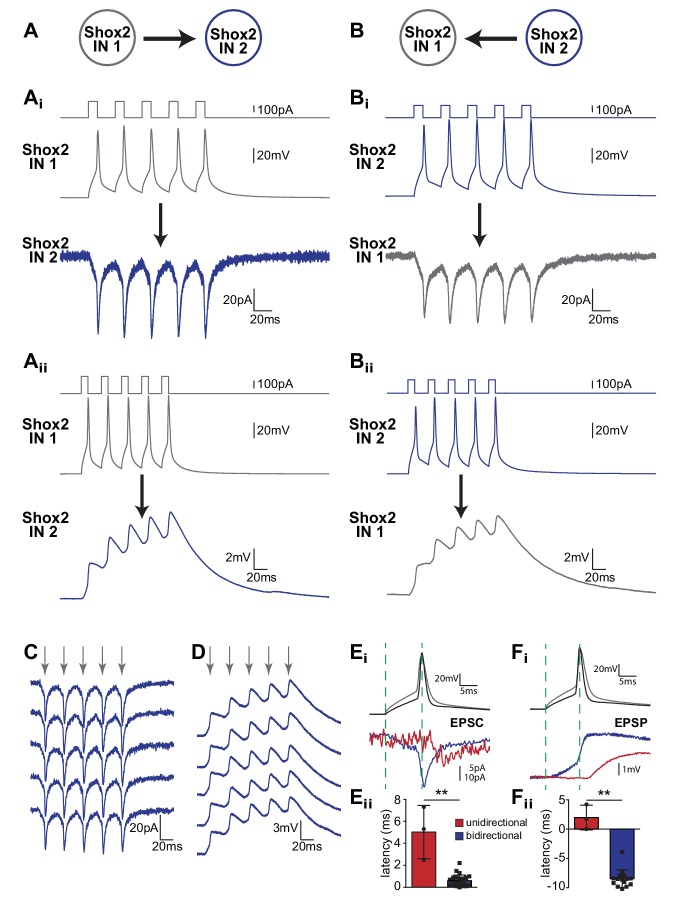
Bidirectional connections are present between Shox2 INs in neonatal spinal cord. (**A** and **B**) Examples of recordings from a pair of bidirectionally connected Shox2 INs. (**Ai** and **Bi**) Cartoon corresponding to colors of traces in (**A** and **B**). (**Ai** and **Aii**) Current injections into Shox2 IN 1 evoked five action potentials (gray). Excitatory postsynaptic currents (**Ai**) or potentials (**Aii**) resulted in Shox2 IN 2 (blue) in voltage clamp or current clamp mode. Reversal of the protocol, current injection in Shox2 IN 2 (blue) also resulted in excitatory currents (**Bi**) or potentials (**Bii**) in Shox2 IN 1. All data in A and B were averages of 50 trials. (**C** and **D**) Examples of individual trials between bidirectionally connected pairs recorded in voltage clamp (**C**) and current clamp (**D**). Gray arrows signify the peaks of the presynaptic action potentials. Note the lack of failures and that responses appear nearly identical in each sweep. (**Ei**) Action potentials evoked in presynaptic unidirectionally (black) and bidirectionally (gray) connected pairs and examples of single postsynaptic currents (red, unidirectional; blue, bidirectional). Dotted lines highlight the beginning of the depolarization and action potential peak in the presynaptic cells, in order to visualize latency differences in the postsynaptic cells. (**Eii**) Mean latency of the EPSC peak, referenced to the peak of the evoked action potential between unidirectionally connected pairs (red) and bidirectionally connected pairs (blue). (**Fi**) Similar to E_i_ but current clamp recordings showing single EPSPs in postsynaptic unidirectional (red) and bidirectional (blue) Shox2 IN pairs in relation to the action potentials in their respective presynaptic Shox2 INs (black and gray). Dotted lines correspond to the start of the depolarization and the peak of the action potential in the presynaptic neurons to highlight the differences in the latency between the two types of connections. (**Fii**) Mean EPSP latency, peak of presynaptic action potential to start of postsynaptic depolarization, is shown for the unidirectional (red) and bidirectional (blue) Shox2 IN pairs. The depolarization in bidirectional pairs precedes the presynaptic action potential, resulting in a negative latency value. ** indicates p<0.01. Error bars represent SD. 10.7554/eLife.42519.004Figure 2—source data 1.Mean latency of EPSC source data for Figure 2E_ii_. 10.7554/eLife.42519.005Figure 2—source data 2.Mean latency of EPSP source data for Figure 2F_ii_.

Using the same protocols described above, we also observed bidirectional coupling between Shox2 INs with a higher incidence than unidirectional connections (n = 59 of 239 pairs, 51 of 155 in slices and 8 of 84 in dorsal horn-removed preparations). Specifically, action potentials evoked by current injection in one Shox2 IN produced EPSCs and EPSPs in the other Shox2 IN and vice versa (Figure 2A and B). Unlike unidirectional connections, a postsynaptic response was present for every action potential in every sweep (Figure 2C and D). The mean latency (average of 50 sweeps) for the postjunctional current, measured peak to peak, was 0.6 ± 0.5 ms (n = 26), which was significantly shorter than that measured in unidirectionally connected pairs (Figure 2E, Mann Whitney, p=0.0058), and likely too short to be mediated by chemical synaptic transmission. The mean peak amplitude of the corresponding postjunctional current was −11.1 ± 9.4 pA (n = 26), which was not statistically different (Mann Whitney, p=0.54) from that measured in unidirectional pairs (−10.6 ± 3.9 pA). In current clamp, it was evident that the postsynaptic EPSPs began prior to the peaks of the presynaptic action potentials, which was in stark contrast with the postsynaptic EPSPs observed in pairs that were connected unidirectionally (Figure 2F). In fact, in bidirectionally-connected pairs, depolarization started −8.5 ± 1.5 ms (n = 15) before the action potential peak, corresponding to the subthreshold depolarization in the current-injected cell. In the unidirectional pairs, the EPSPs began 2.0 ± 2.1 ms (n = 3) after the action potential peak (Mann Whitney, p=0.0092). The mean peak amplitude of the first EPSPs measured in bidirectionally connected pairs was 1.2 ± 1.0 mV (n = 21), which was not different (Mann Whitney, p=0.54) from that in unidirectional pairs (1.4 ± 0.6 mV). Given the bidirectional nature and short latency of this response, we hypothesized that bidirectional connections were mediated by electrical transmission.

### Bidirectionally-connected Shox2 IN pairs are electrically coupled

**Figure 3.**
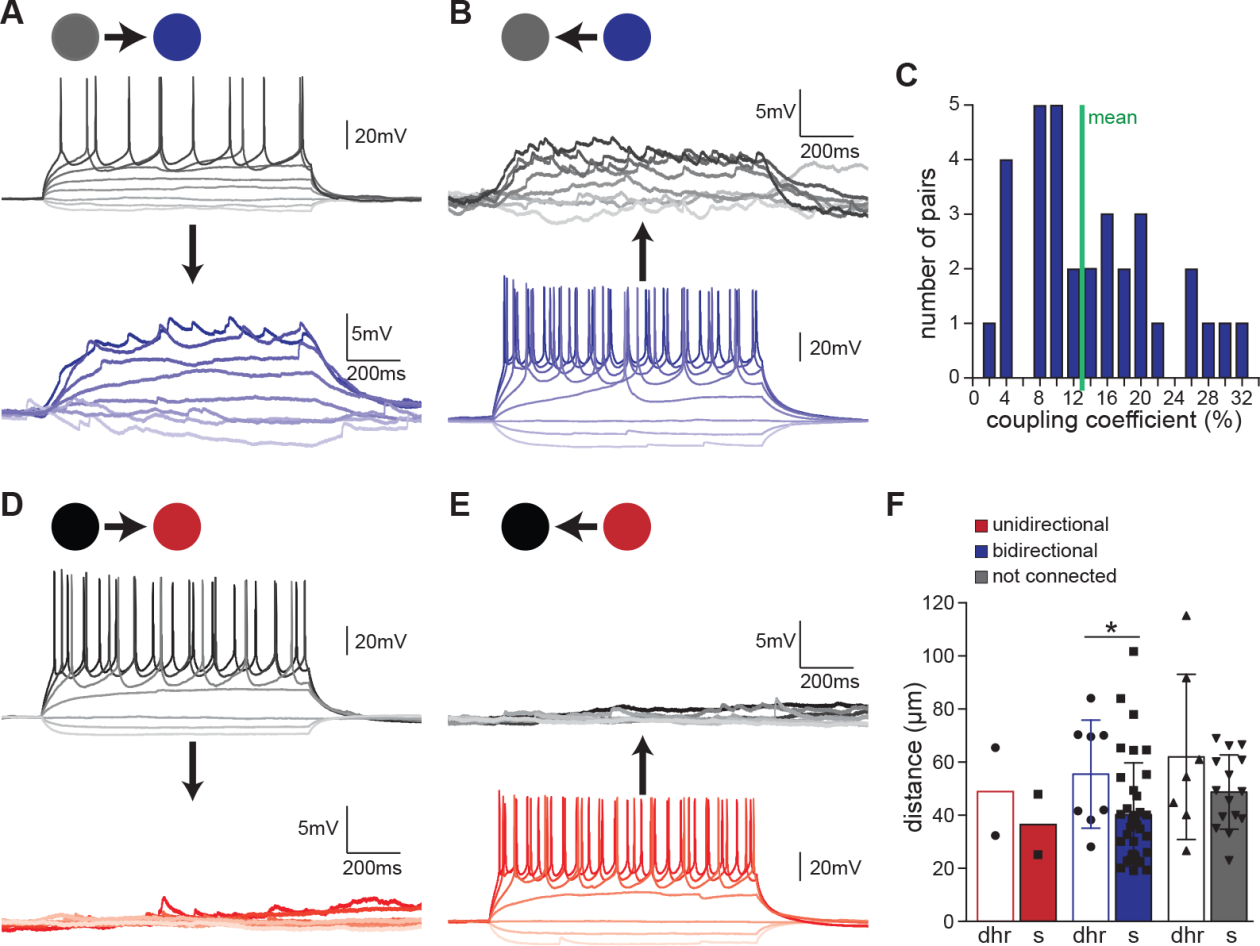
Bidirectional connectivity is due to electrical coupling. (**A** and **B**) Examples of recordings from a pair of bidirectionally connected Shox2 INs. In (**A**), responses of Shox2 IN 1 (gray) and Shox2 IN 2 (blue) to 1 s long hyperpolarizing and depolarizing current steps injected into Shox2 IN one are shown. In (**B**), the protocol was reversed and responses to current steps in Shox2 IN2 are shown. As typical in bidirectionally connected Shox2 INs, both hyperpolarizing and depolarizing responses were evident in the non-injected cell. Additionally, spikelets in the non-injected neuron corresponded to action potentials generated in the neuron receiving the current steps. Darker shading corresponds to increasing current steps. (**C**) Coupling coefficients were highly variable with a mean of 13%, indicated by the green line. (**D** and **E**) Examples of recordings from a pair of unidirectionally connected Shox2 INs. In (**D**), 1 s long current steps were injected into Shox2 IN one while responses of Shox2 IN 1 (black) and Shox2 IN 2 (red) were recorded in current clamp mode. In (**E**), the same protocol was performed but current was injected into Shox2 IN 2. Darker shading corresponds to increasing current steps. (**F**) Distance between recorded neurons was not significantly different by connection type but connected cells were significantly closer together in slices than in dorsal horn-removed preparations. Empty bars for dorsal horn-removed preparations (dhr), filled bars for slices (s), unidirectional (red), bidirectional (blue), or not connected (gray) pairs, mean ±SD. 10.7554/eLife.42519.007Figure 3—source data 1.Coupling coefficients in neonates source data for Figure 3C. 10.7554/eLife.42519.008Figure 3—source data 2.Distance between recorded neurons source data for Figure 3F.

To further test electrical coupling, long (1 s) hyperpolarizing and depolarizing current pulses were injected into Shox2 INs sequentially, while the response was recorded from the other Shox2 IN (noninjected IN). In all Shox2 pairs with bidirectional connections, injection of hyperpolarizing current in one neuron resulted in hyperpolarizing membrane potential in the noninjected IN. Similarly, depolarizing current injected in one Shox2 IN resulted in depolarization of the other. In many cases, action potentials in the presynaptic neuron produced corresponding spikelets in the non-injected cell (Figure 3A and B). Summation of spikelets to generate action potentials was not observed in any of the recorded pairs. Coupling coefficients were also calculated as the response voltage amplitude in the non-injected neuron divided by the voltage amplitude in the injected neuron at the current step prior to rheobase (Figure 3C). Coupling coefficients widely varied, ranging from 2% to 31% with a mean of 13 ± 8% (n = 33), similar to that reported in Hb9 INs (Hinckley and Ziskind-Conhaim, 2006). Transfer of current in both directions strongly suggested that these Shox2 INs were connected electrically. This is in contrast to unidirectional pairs, in which subthreshold current injections to either of the two recorded neurons had no effect on the other neuron (Figure 3D and E).

One possible contributor to the likelihood of a Shox2 IN pair being unidirectional or bidirectional is distance. All somata of Shox2 IN pairs recorded in this study were in close proximity. The average distance between electrically coupled Shox2 INs measured as the distance between the tips of the two recording electrodes was 40 ± 19 µm (n = 37) and 56 ± 20 µm (n = 8) in slices and dorsal horn-removed preparations, respectively. The distances between connected pairs were significantly different in the two preparations but neither was significantly different from the distances between non-connected pairs (49 ± 14 µm, n = 15 in slices; 62 ± 31 µm, n = 7 in dorsal horn removed; two-way ANOVA, Mann-Whitney posthoc, p=0.22 for connected compared to non-connected, p=0.0302 for slices compared to dorsal horn removed connected, p=0.44 for slices compared to dorsal horn in non-connected; Figure 3F). Distances between unidirectional pairs (slices: 25 µm and 48 µm, dorsal horn removed: 32 µm and 65 µm) fell within the range of both electrically connected and non-connected pairs. However, the proximity of the recordings and the way in which we targeted the neurons may have biased our sample toward Shox2 INs more likely to be electrically connected since Shox2 INs were chosen for recordings by the presence of clear processes from one Shox2 IN passing in close proximity to the soma of another Shox2 IN, particularly in pairs recorded in spinal slices. It is expected that distance between neurons will be a factor in the type of connection, with incidence of electrical coupling decreasing at greater distances. However, between Shox2 INs in close proximity, there were no differences in the distances between the observed unidirectional and bidirectional pairs.

### Bidirectional connections may be mediated entirely by gap junctional coupling

**Figure 4.**
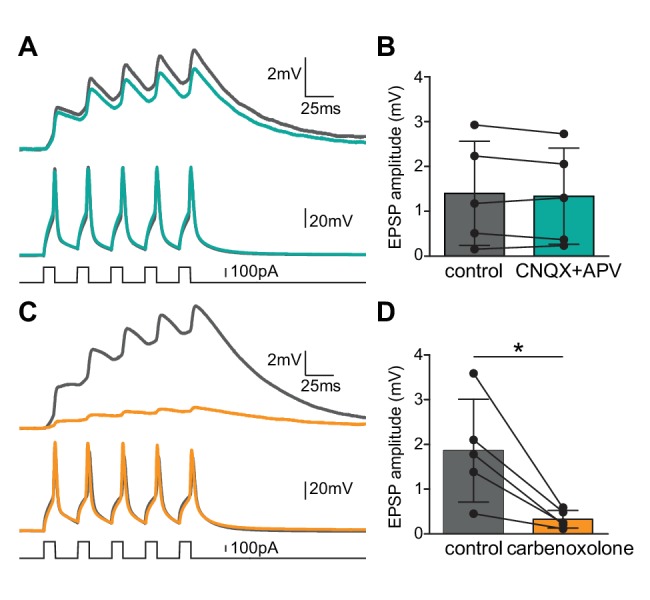
Electrical coupling between Shox2 INs does not have a chemical component. (**A**) Examples of recordings from a bidirectionally connected pair of Shox2 INs. Averaged recordings prior to (gray) and after the addition AMPA receptor and NMDA receptor antagonists, CNQX and APV or CPP (teal). Current injections into Shox2 IN 1 (bottom) elicited action potentials in the stimulated/presynaptic neuron (middle) and depolarizations in Shox2 IN2 (top). (**B**) There was no significant change to the amplitude of the first EPSP when fast glutamatergic transmission was blocked. (**C**) Examples of recordings from a bidirectional connected pair of Shox2 INs prior to (gray) and after the addition of gap junctional blocker, carbenoxolone (yellow). Traces are in the same order as in (**A**). (**D**) Bar graphs showed the amplitude of the first EPSP in the postsynaptic Shox2 IN in response to the first of five action potentials evoked in the presynaptic neuron. Carbenoxolone efficiently decreased the EPSPs in the responding Shox2 IN. * indicates p<0.05. Error bars represent SD. 10.7554/eLife.42519.010Figure 4—source data 1.EPSP amplitude pre- and post-glutamatergic antagonist source data for Figure 4B. 10.7554/eLife.42519.011Figure 4—source data 2.EPSP amplitude pre- and post-carbenoxolone source data for Figure 4D.

Mixed electrical and chemical synapses have been implicated in various systems. As we have identified infrequent presumptive chemical connections between Shox2 INs, we wanted to further investigate whether electrically coupled Shox2 INs showed an additional chemical component. Ionotropic glutamatergic transmission was blocked by bath application of CNQX (10 µM) and CPP (10 µM) or APV (10 µM), AMPA receptor and NMDA receptor antagonists, respectively (Figure 4A). The presence of antagonists did not change the amplitude of first EPSPs (average of 50 sweeps) in electrically coupled Shox2 INs (Figure 4B, control: 1.4 ± 1.2 mV, antagonists: 1.3 ± 1.1 mV, n = 5 pairs, paired t-test, p=0.41). Similar to the first EPSP, the fifth EPSP was not significantly different in the CNQX and APV/CPP (control: 3.3 ± 2.6 mV, antagonists: 2.7 ± 2.1 mV, n = 5 pairs, paired t-test, p=0.12). We further determined the contribution of electrical coupling using a gap junction blocker, carbenoxolone (100 µM). Following 30 min of application, carbenoxolone decreased the first EPSP of each sweep in the non-injected Shox2 IN to just 19 ± 2% of control (Figure 4C and D, control: 1.9 ± 1.1 mV, carbenoxolone: 0.3 ± 0.2 mV, n = 5 pairs, paired t-test, p=0.0242). Similar to the first EPSP, the fifth EPSP was significantly reduced in carbenoxolone (control: 5.2 ± 3.2 mV, carbenoxolone: 1.0 ± 0.7 mV, n = 5 pairs, paired t-test, p=0.0234). In most cases the small remaining EPSPs did not shift in latency indicating that the remaining EPSPs were likely to be electrically, rather than chemically mediated. Altogether, this suggests that bidirectional connections are likely exclusively mediated by electrical synapses.

### Electrical synapses between Shox2 INs act as low-pass filters

**Figure 5.**
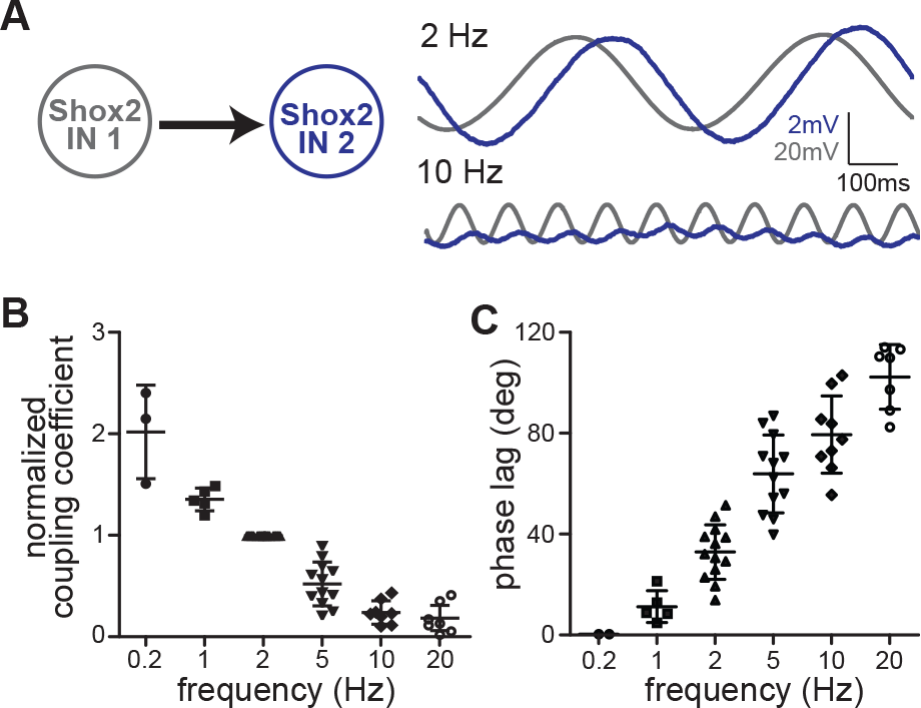
Electrical synapses between Shox2 INs act as low-pass filters. (**A**) Membrane oscillations in Shox2 IN 1 (gray) and Shox2 IN 2 (blue) resulting from subthreshold sinusoidal current injections (±20 pA) to Shox2 IN 1 at 2 Hz and 10 Hz frequencies. All traces are averages of 10 sweeps. (**B**) Coupling coefficients normalized to value at 2 Hz to demonstrate frequency-dependence. Coupling strength decreased with increasing frequency of injected current (0.2 Hz; n = 3; 1 Hz, n = 5; 2 Hz, n = 13; 5 Hz, n = 12; 10 Hz, n = 8; and 20 Hz, n = 7) Error bars represent SD. (**C**) Phase lag is frequency dependent. As the frequency of the injected current increased, phase lag increased (0.2 Hz, n = 2; 1 Hz, n = 5; 2 Hz, n = 13; 5 Hz, n = 12; 10 Hz, n = 9; and 20 Hz, n = 7). 10.7554/eLife.42519.013Figure 5—source data 1.Coupling coefficient source data for Figure 5B. 10.7554/eLife.42519.014Figure 5—source data 2.Phase lag source data for Figure 5C.

**Figure 6.**
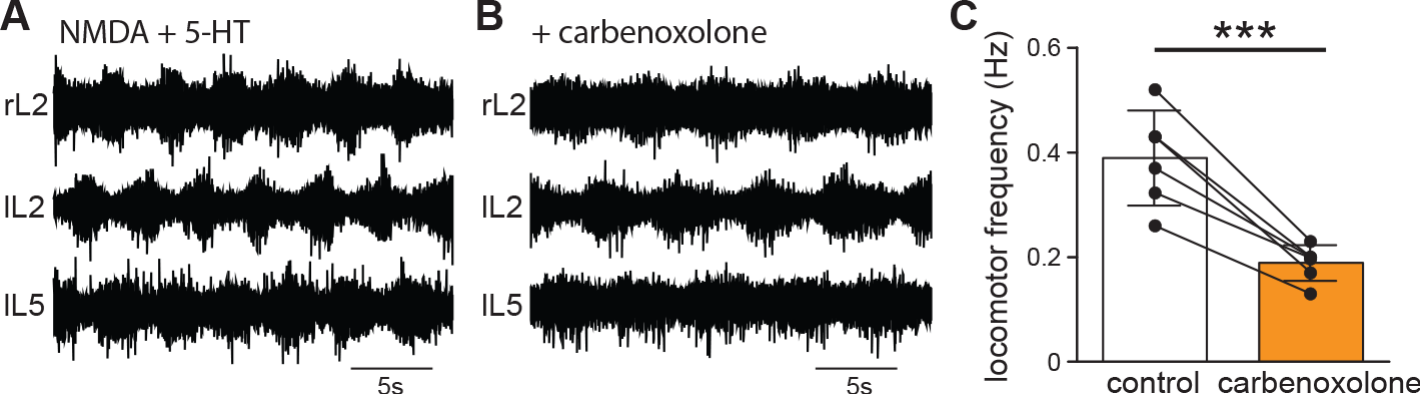
Blocking gap junctions with carbenoxolone decreases locomotor frequency. (**A**) Extracellular recordings from ventral roots at lumbar level 2 (L2)-flexor dominant root- and level 5 (L5)-extensor dominant root- on the right (r) and on the left (l) side of the spinal cord after application of NMDA (7 μM) and serotonin (8 μM). Alternation in ventral root bursts was present between the flexor and extensor root as well as between the right side and the left side of the spinal cord. (**B**) Addition of carbenoxolone (100 μM) decreased the frequency of locomotion (analyzed after 40 min of wash in) with little to no change in the pattern of locomotion. (**C**) Quantification of locomotor frequency shows that it is significantly reduced by the addition of carbenoxolone. *** indicates p<0.005. Error bar represents SD.

Gap junctions are often thought of as low-pass filters (Galarreta and Hestrin, 1999; Gibson et al., 2005; Rekling et al., 2000; Hinckley and Ziskind-Conhaim, 2006). Locomotor-related cellular oscillations are typically low frequency and therefore are likely to be partly transferred through gap junctions. In order to test the strength of electrical coupling between Shox2 INs as a function of frequency, we next injected subthreshold sinusoidal currents at frequencies of 0.2, 1, 2, 5, 10, and 20 Hz while monitoring the changes in membrane potentials both in the injected Shox2 IN and the electrically connected Shox2 IN (Figure 5A). We then measured both the coupling coefficient (Figure 5B) and the phase lag (Figure 5C) that occurred between the injected neuron and the non-injected neuron and found that as the frequency of the injected current increased, the coupling coefficient decreased and the phase lag increased. As frequencies of drug evoked locomotion in neonatal mice range from approximately 0.2–0.8 Hz in isolated spinal cord preparations (Talpalar and Kiehn, 2010), this should place locomotor frequencies within a range where high coupling between Shox2 INs would occur. We reasoned that if electrical coupling played an important role in generating and promoting the rhythm in Shox2 INs, blocking gap junctions with carbenoxolone would have an effect on the frequency of locomotion. In order to test this, we recorded from a flexor-related (lumbar L2 or L3) and an extensor-related (L5) ventral root in isolated spinal cord preparations during locomotor-like activity evoked by NMDA and 5-HT (Figure 6A). Here, we saw that after 40 min of adding carbenoxolone (100 µM) to the bath, locomotor frequency was significantly reduced (control: 0.39 ± 0.09 Hz, carbenoxolone: 0.19 ± 0.03 Hz, n = 6 cords, paired t-test, p=0.0009; Figure 6B and C). Although electrical connections between motor neurons (Tresch and Kiehn, 2000), Hb9 INs (Hinckley and Ziskind-Conhaim, 2006), or other CPG INs (Zhong et al., 2010) are likely to contribute as well and carbenoxolone may have non-specific effects on the network (Rekling et al., 2000; Vessey et al., 2004; Tovar et al., 2009; Connors, 2012), this suggests that electrical coupling between Shox2 INs could act as a mechanism to promote locomotor rhythmicity.

### Shox2 electrical coupling between Shox2 INs declines in incidence and strength with age

**Figure 7.**
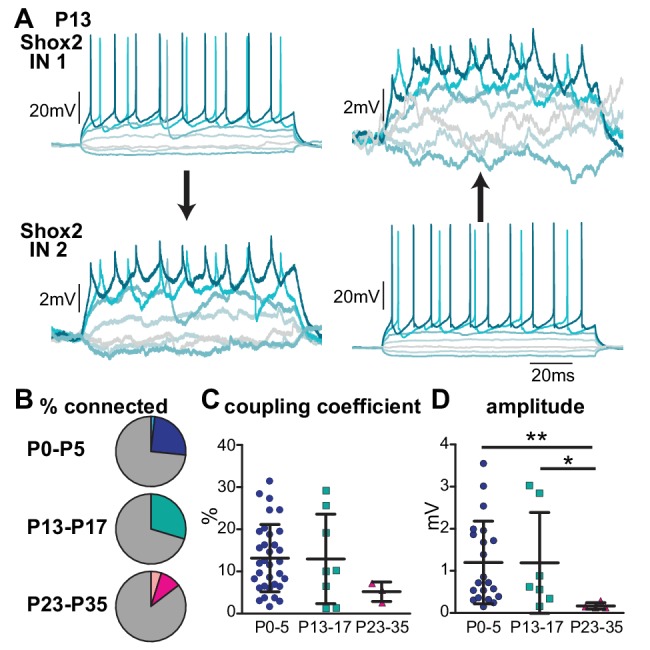
Electrical coupling between Shox2 INs is age-dependent. (**A**) Example of bidirectional electrical coupling detected between Shox2 INs in a P13 mouse. Hyperpolarizing and depolarizing current steps injected into either Shox2 IN 1 (left) or Shox2 IN 2 (right) resulted in hyperpolarizations and depolarizations in both neurons. Shading of lines is to better visualize separate sweeps of differing injected currents. Spikelets were observed in the connected Shox2 INs corresponding to action potentials generated in the IN depolarized by injected current. (**B**) Pie charts indicate the incidence of connectivity between Shox2 INs detected in P0-P5, P13-P17, and P23-P35 age groups. Darker colors in each represent bidirectional connections. Lighter wedges in P0-P5 and P23-P35 represent unidirectional connections. (**C**) Strength of coupling coefficient in bidirectionally connected Shox2 INs from different age groups as the mice mature. (**D**) Amplitude of the EPSP in the postsynaptic Shox2 IN in response to the first of five action potentials evoked in the presynaptic neuron in different age groups. * indicates p<0.05 and ** indicates p<0.01. Error bars represent SD. 10.7554/eLife.42519.017Figure 7—source data 1.Coupling coefficients by age group source data for Figure 7C. 10.7554/eLife.42519.018Figure 7—source data 2.EPSP amplitudes by age group source data for Figure 7D.

Electrical transmission has been shown to be prevalent during early postnatal period, but often decreases as the animal matures. Although there is structural evidence for maintained connexin expression in the adult spinal cord (Rash et al., 1996; Chang et al., 1999; Nagy et al., 2004; Rash et al., 2000; Personius et al., 2007; Marina et al., 2008; Bautista et al., 2012), electrophysiological evidence has yet to be demonstrated. Electrical coupling persists at least to P11 in spinal interneurons expressing Hb9 (Hinckley and Ziskind-Conhaim, 2006). Consequently, we wanted to ask whether electrical transmission in Shox2 INs continued in later stages or is limited to early postnatal mice. Whole cell paired recordings were performed in slices from older mice categorized into three age groups: P13-P17, P23-P35, and adult (>P55). Similar to neonates, electrical coupling was present in 30% of Shox2 IN pairs (n = 8/27) in the P13-17 group (Figure 7A and B). Electrical coupling of Shox2 IN pairs dropped off in incidence to 10% in P23-35 mice (n = 4/41) and was not detected in adult (n = 0/22). The proportions of electrically connected Shox2 INs were significantly different between age groups (chi-square test, p=0.008). Of the pairs that were coupled, the mean electrical coupling coefficient was determined to be 13 ± 11% (n = 8) in the P13-17 mice and 5 ± 2% (n = 3) in P23-35 mice (Figure 7C). Although not statistically different (Kruskal-Wallis, p=0.19), there were few connections detected in the P23-35 group due to the lower incidence rate. The amplitude of EPSPs measured in the postjunctional Shox2 IN in response to the evoked action potentials in the prejunctional Shox2 INs decreased in the P23-35 mice (mean = 0.2 ± 0.1 mV, n = 4) compared to the P0-5 (mean = 1.2 ± 1.0 mV, n = 21) and P13-17 groups (mean = 1.2 ± 1.2 mV, n = 7, Kruskal-Wallis, p=0.0114, Dunn’s Multiple Comparison post-hoc test, P23-35 vs. P0-5, p<0.01,and P23-35 vs. P13-17, p<0.05, Figure 7D). However, the EPSPs in the neonatal group were not significantly different from those measured in the P13-17 group (Dunn’s Multiple Comparison post-hoc test, p>0.05). Overall, this suggests that a reduction in the electrical transmission between Shox2 INs, both in the number of connections and the amplitude of response in the non-injected cell, begins by the third postnatal week.

### Preferential connections exist within different functional groups of Shox2 INs

**Figure 8.**
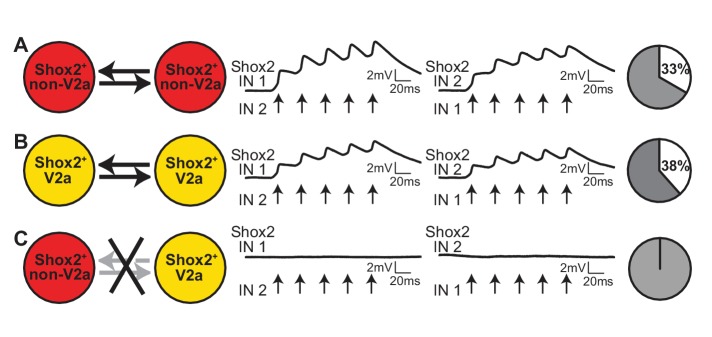
Connectivity between Shox2 INs depends on function. (**A**) Examples of recordings from a pair of Shox2^+^ non-V2a INs. Average of 50 responses of Shox2 IN 1 and Shox2 IN 2 to 5 action potentials evoked with current steps applied to the other IN. Action potential peaks are indicated with arrows. Pie graphs show the proportion of Shox2^+^ non-V2a pairs found to be bidirectionally connected (white) and not connected (gray). (**B**) Example recordings from a pair of Shox2^+^ V2a INs, as in (**A**). (**C**) Example recordings from a mixed pair consisting of one Shox2^+^ non-V2a INs and one Shox2^+^ V2a INs, displayed as in (**A**).

Shox2 INs can be divided into two populations based on the expression of the transcription factor Chx10 (Shox2^+^ V2a INs and Shox2^+^ non-V2a INs). Genetic manipulation experiments have attributed different functions to Shox2^+^ V2a and Shox2^+^ non-V2a INs. Shox2^+^ non-V2a INs are involved in locomotor rhythm generation and Shox2^+^ V2a INs are important for stabilizing motor bursts, likely by providing input to motoneurons (Dougherty et al., 2013). Therefore, we next asked whether interconnections between Shox2 INs are related to functional groupings. For these experiments, we used Shox2::Cre; Chx10::eGFP; Rosa26-lsl-tdTomato mice in order to distinguish Shox2^+^ non-V2a INs (red only) from Shox2^+^ V2a INs (red and green). We then performed paired recordings from 2 Shox2^+^ non-V2a INs, 2 Shox2^+^ V2a INs, or 1 Shox2^+^ non-V2a IN and 1 Shox2^+^ V2a IN (Figure 8). When pairs of Shox2^+^ non-V2a INs were targeted for the recording, 33% were electrically coupled (n = 6 of 18 pairs). Similarly, when pairs of Shox2^+^ V2a INs were targeted, 38% were electrical connected (n = 5 of 13 pairs). However, when mixed pairs of Shox2^+^ non-V2a INs with Shox2^+^ V2a INs were recorded, we were not able to detect any connections (n = 0 of 12 pairs). Altogether this suggests gap junctional interconnectivity is preferential within functional groupings of Shox2 INs.

## Discussion

Our present study explored interconnectivity within the Shox2 IN population, part of which has been proposed to contribute to locomotor rhythm generation. Two types of connections between Shox2 INs were identified in neonatal mice. Unidirectional connections, consistent with chemical synapses, were found at a very low incidence rate. Far more common were bidirectional connections mediated by gap junctions. Functional gap junctional coupling between Shox2 interneurons continued into more mature mice that are capable of weight supported stepping but began to decrease in incidence and strength around the third postnatal week and was not detectable in adult. Electrical coupling was preferential within functional groupings of Shox2 INs as separated by the presence or absence of the transcription factor Chx10. Thus, gap junctional coupling provides a potential mechanism for synchronous activation of rhythm-generating neurons, particularly in young animals.

### Shox2 INs are interconnected by electrical synapses

We found that nearly a third of Shox2 INs were electrically coupled and a very small percentage of Shox2 INs were chemically coupled. Gap junctional connections are most likely to occur between nearby neurons as connexin 36 has been shown to be located on somata and proximal dendrites (Rash et al., 2001; Marina et al., 2008; Bautista et al., 2012). Here, the somata of Shox2 INs in paired recordings were typically within ~65 μm of each other and those with processes running between them were preferentially chosen for recordings. The way in which the pairs were targeted likely contributes to the relatively high degree of electrical coupling which was not seen in previous paired recordings where processes were not traced (Dougherty et al., 2013). Consequently, by testing local interneurons, we are likely biasing our sample towards a higher degree of electrical connectivity. In contrast, it is possible that the low incidence of chemically-mediated connections detected between Shox2 INs is an underestimate since we restricted our sampling to local interneurons and slicing disrupts axonal projections and dendrites. Sparse recurrent connections within excitatory populations can support locomotor rhythm generation in computational models of rodent central pattern generators (Zhong et al., 2012; Shevtsova et al., 2015; Bui and Brownstone, 2015; Shevtsova and Rybak, 2016; Ausborn et al., 2018) and are thought to underlie rhythm generation in other networks (Grillner, 2003; Li et al., 2006; Kozlov et al., 2007), and this is even more robust when both electrical and reciprocal synapses are present (Asghar et al., 2005; Li et al., 2009; Hull et al., 2015). Here, similar to rhythmogenic respiratory neurons in the pre-Bötzinger complex, none of the chemically coupled rhythmogenic Shox2 neurons were electrically coupled and vice versa (Rekling et al., 2000). Although it is possible that the remaining potential seen in carbenoxolone is due to mixed chemical synapse, carbenoxolone does not completely block gap junctions (Kiehn and Tresch, 2002). Further, the time courses of the remaining potentials match with those mediated by gap junctions and do not appear to have a synaptic delay. Although we did not detect any pairs that were both electrically and chemically connected, we do not think that one connection necessarily precludes the other. Chemical connections were found at too low of an incidence to rule out the possibility that some electrically coupled neurons are also chemically connected.

We also observed a high failure rate in synaptic transmission in chemically-mediated Shox2 IN connections. This would be expected if the connections are not monosynaptic and we cannot rule out this possibility. Nevertheless, synaptic failures in unitary connections have been described in other regions of the central nervous system (Bolshakov and Siegelbaum, 1995; Stevens and Wang, 1995; Rekling et al., 2000) and high failure rates are linked to an enhanced propensity for long-term potentiation (Marina et al., 2008; Bolshakov and Siegelbaum, 1995). Thus, modifying synaptic fidelity between Shox2 neurons, monosynaptically or disynaptically connected, should be possible based on development, activity dependent mechanisms, or neuromodulatory control (Bolshakov and Siegelbaum, 1995; Chang et al., 1999; Chang et al., 1999; Mentis et al., 2002; O'Brien, 2014; Marder et al., 2017). These possibilities are not mutually exclusive and raise interesting mechanisms to explore in terms of rhythm generation and dynamic circuit connectivity of the locomotor network.

### Functional groups of Shox2 INs are preferentially connected

Using a triple transgenic strategy, we were able to distinguish between the two known functional populations of Shox2 INs. Approximately 1/4 of the Shox2 IN population does not express Chx10 (Shox2^+^ non-V2a) and is thought to be part of the locomotor rhythm generator. The remaining Shox2 INs (~3/4 of population) co-express the transcription factor Chx10 (Shox2 V2a IN) and are proposed to belong to the pattern forming layer of the CPG, providing input to motoneurons (Dougherty et al., 2013). Shox2 IN interconnections were preferential within each subpopulation (Shox2^+^ non-V2a to Shox2^+^ non-V2a and Shox2^+^ V2a to Shox2^+^ V2a). Electrical connections have previously been shown within the Chx10 population (not divided by Shox2 expression) in neonatal (P1-4) mice and coupling incidence was found to be higher among neurons with the same firing properties (Zhong et al., 2010). Shox2 INs (V2a and non-V2a) display the similar firing properties to those reported in the Chx10 population (Dougherty and Kiehn, 2010; Zhong et al., 2010). Therefore, these two populations cannot be simply split by electrophysiological signatures; however, when considering subpopulations based on both molecular markers and firing properties, connectivity rates may be even higher. Similar separations by function have been previously seen both in motor neurons, where only homonymous pools are electrically connected (Walton and Navarrete, 1991; Kandler and Katz, 1995), in Hb9 neurons, where GFP populations were only electrically connected when they still contained Hb9 protein (Hinckley and Ziskind-Conhaim, 2006), and in descending rhythm-generating interneurons in Xenopus, which are not electrically coupled with other CPG neurons (Li et al., 2009), however, this is not always the case (Eisen and Marder, 1982; Wilson et al., 2007; Chopek et al., 2018). It is also possible that the presence/absence or range in strength of electrical coupling in Shox2 neurons is related to a further subdivision in transcription factor expression (i.e. Hayashi et al., 2018) or function (i.e. phasing relationship) of Shox2 INs. Altogether, preferential interconnectivity within molecularly-defined subsets of Shox2 INs further supports distinct functional roles of these populations.

### Functional implications of interconnectivity between Shox2 INs

Gap junctional coupling is highly effective at promoting synchronization in neuronal activities (Kiehn and Tresch, 2002; Personius et al., 2007; Wilson et al., 2007; Zhong et al., 2010) and has been implicated in rhythm generation (Marder and Calabrese, 1996; Rekling et al., 2000; Tresch and Kiehn, 2000; Bou-Flores and Berger, 2001; Sharifullina et al., 2008; Li et al., 2009; Pierce et al., 2010). Additionally, gap junctional coupling allows subthreshold activities to contribute to network function (Marder and Calabrese, 1996). Therefore, a network with prominent gap junctional coupling would require less extrinsic drive to initiate synchronous rhythmic oscillations, and this drive could be localized to a part of the population but evoked activity could spread through the network. This would allow for a small number of active neurons to rapidly synchronize specific Shox2 subpopulations. Further, the small number of neurons activated initially would not have to be the same neurons cycle to cycle, in order to result in synchronous activation of the population, as has been recently shown in respiration (Carroll and Ramirez, 2013; Kam et al., 2013; Feldman and Kam, 2015; Del Negro et al., 2018). In terms of locomotor network activity, blocking gap junctional coupling with carbenoxolone decreased the frequency of drug evoked locomotion, consistent with previous findings (Tresch and Kiehn, 2000; Falgairolle et al., 2017). This result is remarkably similar to the effect of removing the population of Shox2 neurons from the network (Dougherty et al., 2013). Thus, it is possible that electrical connections between functionally-related Shox2 INs synchronize neuronal activity and may contribute to rhythmicity. These results should be cautiously interpreted, however, as there are alternative explanations. Gap junctions have been demonstrated between several interneuronal populations (Hinckley and Ziskind-Conhaim, 2006; Wilson et al., 2007; Zhong et al., 2010) and between motor neurons (Walton and Navarrete, 1991; Kandler and Katz, 1995; Rash et al., 1996; Chang et al., 1999; Tresch and Kiehn, 2000; Mentis et al., 2002; Marina et al., 2008). Additionally, functional electrical coupling has been reported between motor neurons and spinal excitatory interneurons in zebrafish (Bhatt et al., 2007; Song et al., 2016) and mouse (Chopek et al., 2018). Lastly, carbenoxolone has been shown to have several non-specific effects, including decreasing input resistance, voltage gated Ca^2+^ currents, and AMPA receptor-mediated currents (Rekling et al., 2000; Vessey et al., 2004; Tovar et al., 2009), which may also lead to similar effects.

Taken together, electrical coupling is prevalent in neonatal mice and preferential to functional groups of Shox2 interneurons. In addition to synchronizing functional populations, it is possible that this serves to promote rhythm generation and/or strengthen connections to downstream targets, i.e. via Hebbian mechanisms (Walton and Navarrete, 1991). Our results demonstrate that electrical coupling persists well through the transition to weight bearing stepping but could not be detected in the adult. This suggests that electrical transmission can serve as one of the mechanisms to synchronize rhythm-generating neurons during spinal circuit development, although this declines with age. In adult animals, it is possible that there is a shift in the neuronal populations requiring synchronization and that Shox2 neurons no longer play this role. We favor the possibility that as connectivity structures mature, other neuronal properties develop that can support similar functions but through different mechanisms. For example, sparse connectivity together with enhancement of intrinsic excitability or neuromodulatory control (Husch et al., 2015) may be sufficient to maintain synchronization and network function. Currently, this is speculative and requires direct experimental testing.

## Materials and methods

**Key resources table:** 

Reagent type (species) or resource	Designation	Source or reference		Identifiers	Additional information	
Genetic reagent (*M. musculus*)	Shox2::Cre	PMID: 24267650				
Genetic reagent (*M. musculus*)	Rosa26-lsl- tdTomato	Jackson Laboratory		Stock #: 007909	PMID: 20023653	
Genetic reagent (*M. musculus*)	Chx10GFP	Mutant Mouse Regional Resource Center		MMRRC Cat#: 011391-UCD	Now called Vsx2-EGFP; PMID: 14586460	
Chemical compound, drug	carbenoxolone disodium salt	Sigma		C4790		
Chemical compound, drug	5-HT, serotonin creatinine sulfate monohydrate	Sigma		H7752		
Chemical compound, drug	NMDA, N-Methyl- D-aspartic acid	Sigma		M3262		
Chemical compound, drug	CNQX, 6-cyano-7- nitroquinoxaline-2,3- dione disodium salt	Tocris		1045		
Chemical compound, drug	AP-5, 2-amino-5- phosphopentanoic acid	Tocris		1234		
Chemical compound, drug	CPP, 3-((R)−2- Carboxypiperazin -4-yl)-propyl-1- phosphonic acid	Tocris		2411		

Experiments were performed using Shox2::Cre (Dougherty et al., 2013); Rosa26-flox-Stop-flox-tdTomato (Ai9 from Jax Mice, #007909, Madisen et al., 2010) or in Shox2::Cre; Ai9; Chx10eGFP (also called Vsx2-eGFP, MMRRC, 011391-UCD, Gong et al., 2003) transgenic mice. All experimental procedures followed NIH guidelines and were approved by the Institutional Animal Care and Use Committee at Drexel University.

### Spinal cord preparations

Spinal cords were isolated as previously described (Dougherty and Kiehn, 2010). Briefly, neonatal (P0-P5) mice were decapitated and eviscerated. Spinal cords were then removed in ice cold dissecting solution containing in mM: 111 NaCl, 3 KCl, 11 glucose, 25 NaHCO_3_, 3.7 MgSO_4_, 1.1 KH_2_PO_4_, and 0.25 CaCl_2_. For experiments in a dorsal horn-removed preparation, the dorsal lumbar region (L2-L5) was subsequently removed from one side of the cord with a surgical knife in order to gain access to the Shox2 INs. For slice experiments, lumbar spinal cord (L2-5) was sectioned transversely (300–350 µm) with a vibrating microtome (Leica Microsystems). Dorsal horn-removed preparations and slices were next transferred to room temperature (RT) artificial cerebrospinal fluid (ACSF) recording solution containing in mM: 111 NaCl, 3 KCl, 11 glucose, 25 NaHCO_3_, 1.3 MgSO_4_, 1.1 KH_2_PO_4_, and 2.5 CaCl_2_. Cords and slices were incubated for at least 30 min prior to recordings. Dissecting and recording solutions were continuously aerated with 95%/5% CO_2_/O_2._ Slices from older mice (P13-P17, P23-P35, and >P55) were obtained in a similar manner with the following differences. Mice > P5 were anesthetized with a mixture of ketamine (150 mg/kg) and xylazine (10 mg/kg) prior to decapitation. Cords were isolated in ice cold glycerol-based ACSF solution containing in mM: 3 KCl, 11 glucose, 25 NaHCO_3_, 1.3 MgSO_4_, 1.1 KH_2_PO_4_, 2.5 CaCl_2_, 222 glycerol. Following sectioning, slices were transferred to ACSF at 37°C for 30 min and then passively equilibrated to RT for another 30 min before recording.

### Patch-clamp recordings

All recordings were performed at room temperature. Fluorescently labeled (tdTomato) Shox2 INs were visualized with a 63X objective lens on a BX51WI scope (Olympus) using LED illumination (X-cite). Patch electrodes were pulled to tip resistances of 5–8 MΩ using a multi-stage puller (Sutter Instruments) and were filled with intracellular solution which contained in mM: 128 K-gluconate, 10 HEPES, 0.0001 CaCl2, one glucose, 4 NaCl, 5 ATP, and 0.3 GTP. In some experiments, biocytin (2 mg/ml, Sigma) was included in the patch electrode. Cells were targeted based on fluorescence and using differential interference contrast (DIC) optics for pairwise whole-cell patch recordings. Recordings were made from pairs of neurons located in close proximity and those with processes appearing to pass the soma of another cell were preferentially targeted. Data was collected with a Multiclamp 700B amplifier (Molecular Devices) and Clampex software (pClamp9, Molecular Devices). Signals were digitized at 10 kHz and filtered at 6 kHz.

To test for connectivity between two patched Shox2 INs, a train of five short (10 ms) strong (100–300 pA) current pulses (interstimulus interval of 20 ms), were applied to the Shox2 IN recorded with electrode 1, ‘Shox2 IN 1’, while the resulting synaptic activity in the other Shox2 IN was recorded with the other electrode, ‘Shox2 IN 2’. This was repeated for a total of 50 sweeps, with a start-to-start interval of 2 s, for offline averaging. In current clamp, biased current was applied to ‘Shox2 IN 2’ so that its membrane potential was around −65 mV. In voltage clamp, ‘Shox2 IN 2’ was held at −60 mV. The same protocol was then reversed and applied from ‘Shox2 IN 2’ to ‘Shox2 IN 1’. Any sweep in which a current pulse failed to produce an action potential in the injected cell was removed prior to averaging which resulted in 25–50 sweeps being averaged for each test. Additionally, series of hyperpolarizing and depolarizing steps were run sequentially to determine firing properties, membrane properties, and electrical connectivity. Coupling coefficient (k) was calculated using the depolarizing current step prior to rheobase and defined as the ratio of the voltage response in the postsynaptic cell to the presynaptic cell. Rheobase was defined as the lowest current step (in 5 pA increments) that evoked an action potential in Shox2 INs. Not every protocol was run for all pairs of neurons. For many of the pairs, either the voltage clamp or the current clamp protocol was run to test for connections. Both were run in later recordings. The protocol to determine coupling coefficient was added partway through the study. In cases where one of the two cells was lost or recording integrity declined, the protocol was not included in analyses. Data from dorsal horn-removed experiments was included in the analysis of incidence, amplitude, and latency. Pharmacology, coupling coefficients, and >P5 experiments were exclusively performed in slices. Following the paired recording, an image of electrode position was captured. Distance between pairs was estimated by measuring the distance between the tips of the electrodes from the image file. An image was not collected for nine connected pairs and we began capturing images for non-connected pairs late in the study. If there was no image, the pairs were not included in the distance analysis.

### Ventral root recordings

Ventral root activity (signal band-pass filtered 10–1,000 Hz; gain 1000) was recorded from two lumbar (L) 2–5 ventral roots with tightly-fitting glass suction electrodes. The combination of N-Methyl-D-aspartic acid (NMDA, 7 µM, Sigma) and serotonin creatinine sulfate monohydrate (5-HT, 8 µM, Sigma) was bath applied to induce fictive locomotion.

### Pharmacology

In some experiments, fast glutamatergic synaptic transmission was blocked with AMPA receptor antagonist, 6-cyano-7-nitroquinoxaline-2,3-dione disodium salt (CNQX, 10 µM, Tocris), and NMDA receptor antagonist, 2-amino-5-phosphopentanoic acid (AP-5, 10 µM, Tocris) or 3-((*R*)−2-Carboxypiperazin-4-yl)-propyl-1-phosphonic acid (CPP, 10 µM, Tocris). In other experiments, the gap junction blocker carbenoxolone (100 µM, Sigma) was applied.

### Statistics

Statistical tests and post-hoc analyses used are stated for each experiment. All results are presented as mean ±SD. Statistical significance was set at p<0.05.

## Additional files

Transparent reporting form

### Data availability

All data generated or analyzed during this study are included in the manuscript and supporting files. Source data files have been provided for Figures 2Eii, 2Fii, 3C, 3F, 4B, 4D, 5B, 5C, 7C and 7D.
