# Supplementary material for: Spinal Shox2 interneuron interconnectivity related to function and development
Source: eLife. 2018 Dec 31;7:e42519. doi: 10.7554/eLife.42519 (PMC6333440; doi:10.7554/eLife.42519)
Supplement: Transparent reporting form [file elife-42519-transrepform.docx]

***eLife’s* transparent reporting form**

We encourage authors to provide detailed information *within their submission* to facilitate the interpretation and replication of experiments. Authors can upload supporting documentation to indicate the use of appropriate reporting guidelines for health-related research (see [EQUATOR Network](http://www.equator-network.org/%20)), life science research (see the [BioSharing Information Resource](https://biosharing.org/)), or the [ARRIVE guidelines](http://www.plosbiology.org/article/info:doi/10.1371/journal.pbio.1000412) for reporting work involving animal research. Where applicable, authors should refer to any relevant reporting standards documents in this form.

If you have any questions, please consult our Journal Policies and/or contact us: [editorial@elifesciences.org](mailto:editorial@elifesciences.org).

**Sample-size estimation**

- You should state whether an appropriate sample size was computed when the study was being designed
- You should state the statistical method of sample size computation and any required assumptions
- If no explicit power analysis was used, you should describe how you decided what sample (replicate) size (number) to use

Please outline where this information can be found within the submission (e.g., sections or figure legends), or explain why this information doesn’t apply to your submission:

Power analyses were not explicitly used. Our initial sample size estimation was based on expected sparse (5-10%) connectivity of chemical synaptic connections. As we found chemical synapses less frequent than expected (~2%), we were able to obtain a rather large sample of bidirectional connections in the process, which became the focus of the study. In some experiments, the sample size was small (i.e. pharmacology experiments) but the responses were uniform. In these cases, all data points are shown in the figure so that the uniformity in the response is evident to the reader as displayed.

**Replicates**

- You should report how often each experiment was performed
- You should include a definition of biological versus technical replication
- The data obtained should be provided and sufficient information should be provided to indicate the number of independent biological and/or technical replicates
- If you encountered any outliers, you should describe how these were handled
- Criteria for exclusion/inclusion of data should be clearly stated
- High-throughput sequence data should be uploaded before submission, with a private link for reviewers provided (these are available from both GEO and ArrayExpress)

Please outline where this information can be found within the submission (e.g., sections or figure legends), or explain why this information doesn’t apply to your submission:

The number of pairs is mentioned for each experiment within the Results section and the number of sweeps run for each pair is stated in the Methods. In the Methods, we point out that the reasons for variations in the number of pairs between tests (i.e. one of the cells from the pair was not held long enough to complete the protocol for a particular test). All data are included in the calculation of the means and statistics, unless explicitly mentioned in the Results section (i.e. “only sweeps with a response to the first action potential were included in the mean” for Figure 2).

**Statistical reporting**

- Statistical analysis methods should be described and justified
- Raw data should be presented in figures whenever informative to do so (typically when N per group is less than 10)
- For each experiment, you should identify the statistical tests used, exact values of N, definitions of center, methods of multiple test correction, and dispersion and precision measures (e.g., mean, median, SD, SEM, confidence intervals; and, for the major substantive results, a measure of effect size (e.g., Pearson's r, Cohen's d)
- Report exact p-values wherever possible alongside the summary statistics and 95% confidence intervals. These should be reported for all key questions and not only when the p-value is less than 0.05.

Please outline where this information can be found within the submission (e.g., sections or figure legends), or explain why this information doesn’t apply to your submission:

Statistical analyses used are stated in the Methods and relevant Results sections. Raw data are included in figures, when feasible. For graphs generated from datasets with larger Ns, files with the raw data values and calculated means are included as separate source data files (please see below).

(For large datasets, or papers with a very large number of statistical tests, you may upload a single table file with tests, Ns, etc., with reference to sections in the manuscript.)

**Group allocation**

- Indicate how samples were allocated into experimental groups (in the case of clinical studies, please specify allocation to treatment method); if randomization was used, please also state if restricted randomization was applied
- Indicate if masking was used during group allocation, data collection and/or data analysis

Please outline where this information can be found within the submission (e.g., sections or figure legends), or explain why this information doesn’t apply to your submission:

N/A

**Additional data files (“source data”)**

- We encourage you to upload relevant additional data files, such as numerical data that are represented as a graph in a figure, or as a summary table
- Where provided, these should be in the most useful format, and they can be uploaded as “Source data” files linked to a main figure or table
- Include model definition files including the full list of parameters used
- Include code used for data analysis (e.g., R, MatLab)
- Avoid stating that data files are “available upon request”

Please indicate the figures or tables for which source data files have been provided:

Source data files are provided for graphs in the following: Figure 2Eii (EPSC latency, Figure 2Fii (EPSP latency), Figure 3C (coupling coefficient neonates), Figure 3F (distance), Figure 4B (EPSP amplitude pre- and post-glutamatergic antagonists), Figure 4D (EPSP pre- and post-carbenoxolone), Figure 5B (coupling coefficients for different frequencies of currents injected), Figure 5C (phase lag at different frequencies of injected currents), Figure 7C (coupling coefficients by age), and Figure 7D (EPSP amplitude by age).
