# Supplementary material for: Spinal Shox2 interneuron interconnectivity related to function and development
Source: eLife. 2018 Dec 31;7:e42519. doi: 10.7554/eLife.42519 (PMC6333440; doi:10.7554/eLife.42519)
Supplement: Peer review history

# Decision letter

**DOI:** 10.7554/eLife.42519.022

**Affiliations:** Emory UniversityUnited States

In the interests of transparency, eLife includes the editorial decision letter and accompanying author responses. A lightly edited version of the letter sent to the authors after peer review is shown, indicating the most substantive concerns; minor comments are not usually included.

Thank you for submitting your article "Spinal Shox2 interneuron interconnectivity related to function and development" for consideration by *eLife*. Your article has been reviewed by three peer reviewers, including Ronald Calabrese as the Senior/Reviewing Editor and Reviewer #1. The following individuals involved in review of your submission have also agreed to reveal their identity: Robert M Brownstone (Reviewer #2); Ronald M Harris-Warrick (Reviewer #3).

The reviewers have discussed the reviews with one another and the Reviewing Editor has drafted this decision to help you prepare a revised submission.

Summary:

The authors present an elegant electrophysiological analysis of connectivity between Shox2 INs in neonatal and post-natal mouse spinal cord slices. They find a high incidence of bidirectional electrical connectivity and a much lower incidence of unidirectional, failure prone, chemical connectivity (excitatory). They present extensive electrophysiological evidence supporting the electrical connectivity. They show that electrical connectivity declines over the first postnatal month and is apparently absent in adults. They also show that within the Shox2 IN population differentiated by Chx10+/- that electrical connectivity is subclass specific. This is an important observation because previous work has demonstrated that these two classes are involved in pattern (Chx10+) and rhythm (Chx10-) generation respectfully.

The data presented is convincing and clean. These types of detailed studies are necessary to crack the problem of how locomotory rhythms are generated in the spinal cord and how they mature during post-natal development.

Essential revisions:

1) The writing in Introduction and Discussion should be cleaned up and made more concise. Eliminating redundancy, especially in Discussion, will make the paper more readable and impactful.

2) The expert reviewers expressed concern that the conclusion that the chemical unidirectional connections may not indeed be monosynaptic.

3) The Discussion of the role of electrical coupling in rhythmogenesis in the neonate is interesting, but the authors do not show that the electrical coupling is responsible for rhythm generation – would need a specific knock-out to do so, and caution is advised. The idea that electrical coupling is replaced by something else in the adult also needs some caution.

4) There is a major concern that some data is presented as mean and SEM. We request that the data be presented with all points illustrated as in Figure 6C with mean sd or in whisker barrel format.

5) Several major comments from the expert reviewers are appended to amplify these primary concerns.

A) Carbenoxelone: unfortunately, we do not have a specific and reversible gap junction blocker, with Cbx being as good as it gets. Nonetheless, the authors should take care in pointing out the caveats (e.g. calcium channel blocking). Also, did they try it in any unidirectionally/chemically connected pairs? This might help to tease out non-GJ-related effects. Also, on a more minor note, how long was the Cbx applied before effects were seen (subsection “Bidirectional connections may be mediated entirely by gap junctional coupling”)?

B) The preparation: it seems that the authors have done experiments both in slices (I assume transverse but they should state this) and dorsal horn removed preps. It is unclear which experiments were which – I think there should be clarity here (throughout the manuscript, but see, for example, the last paragraph of the Introduction – I can't tell what's what). Also, how deep were the neurons in the latter preparation? And do we know anything about the path that their axons take? Subsection “Unidirectional connections between spinal Shox2 INs are sparse”, end of first paragraph – they expect the connections to be preserved but what's the evidence? Also, were the distances in connectivity different in the different preps? It sounds like the n's aren't high enough to know, but this should all be clear.

C) Chemical transmission: on the above note, of course it is difficult to prove something doesn't exist. I could certainly imagine that these neurons are connected with one another over longer distances, which could be evident in the dorsal horn removed prep, or in horizontal slices. How is there inter-segmental coordination of rhythm, for example? So I think some rewording acknowledging this – i.e. local connectivity only was tested – would be helpful.

D) It is not clear to me that the chemical transmission is monosynaptic. (On that note what temperature were the preps?) How can the authors be sure these aren't disynaptic, thus explaining the low incidence in slice as well as the failures? If they are disynaptic, a whole section of the Discussion can be reduced. (Related: subsection “Unidirectional connections between spinal Shox2 INs are sparse”, third paragraph – why is the EPSP so much faster than the EPSC?)

E) The authors in several locations discuss examples of coupling between like neurons. But this is not always the case in nervous systems, and in fact 100s of millions of years ago, evolution led to coupling of heterogeneous populations of neurons – which has some significant advantages. This has been seen in many invertebrate systems (see crustacean STG work from Marder lab (Eisen and Marder, 1982), for example), and in the mouse spinal cord (see Wilson, Cowan and Brownstone, 2007).

F) Statistics: I have only one issue with their reporting. I will say (as I always do) that the SEM should be eliminated from neuroscience. It is completely meaningless. We are interested in biological variability, which is shown by the standard deviation. We are not particularly interested in mean values, which are what the SEM reflects. It's great that the raw data are shown in places, but it should always be shown, and I would suggest showing either the s.d. (weaker) or using box-whisker plots on top of the raw data (stronger). And in the first paragraph of the subsection “Shox2 electrical coupling between Shox2 INs declines in incidence and strength with age”: there is no tendency: the statistical test showed that the null hypothesis – that they're the same – cannot be rejected. That's why we use statistics.

G) Conceptual model: It would be nice to understand in the Discussion what the authors are proposing. There are a lot of vague sentences like "intriguing functional consequences." I would suggest they explicitly present a conceptual model. (A computational model would be nice, but that's for the next paper, I would imagine!)

# Reply: Author response

**DOI:** 10.7554/eLife.42519.023

Essential revisions:

1) The writing in Introduction and Discussion should be cleaned up and made more concise. Eliminating redundancy, especially in Discussion, will make the paper more readable and impactful.

We have edited the Introduction and, particularly, the Discussion in order to eliminate redundancies pointed out by the reviewers in this general comment and in specific comments below. The Discussion has been extensively reorganized and the length has been significantly reduced.

2) The expert reviewers expressed concern that the conclusion that the chemical unidirectional connections may not indeed be monosynaptic.

We think that the unidirectional connections are monosynaptic; however, we do not have definitive evidence and thus, we agree that we cannot be certain. The specific points made by the reviewers in comments below are valid and all are possible. Therefore, we have removed any assertion that the connections are monosynaptic and have explicitly stated in both the Results and the Discussion that they may not be monosynaptic connections. We do not think that the chemical unidirectional connections being monosynaptic or disynaptic change any of our overall conclusions.

3) The Discussion of the role of electrical coupling in rhythmogenesis in the neonate is interesting, but the authors do not show that the electrical coupling is responsible for rhythm generation – would need a specific knock-out to do so, and caution is advised. The idea that electrical coupling is replaced by something else in the adult also needs some caution.

We have not shown that electrical coupling, particularly that between Shox2 neurons, is responsible for rhythm generation. Data from our experiments suggest that electrical coupling may be implicated in rhythm generation but we agree that caution is necessary in the interpretation. We have adjusted these statements in the Discussion accordingly, adding in additional references to caveats and other possibilities. We have also toned down and clarified the statement regarding electrical coupling being replaced in the adult. We show that electrical coupling decreases in both incidence and strength in older preparations (and is not detected in adult). The point we were attempting to make was that the roles electrical coupling play in synchronization, if necessary in the adult, would be carried out by another mechanism (or another population). We hope this has been made clear in our edited version.

4) There is a major concern that some data is presented as mean and SEM. We request that the data be presented with all points illustrated as in Figure 6C with mean sd or in whisker barrel format.

All graphs have been edited and reported values in the text have been changed to mean +/- SD. All data is now shown as individual data points as mean +/- SD. Mean +/- SD was chosen so that it corresponds directly with the text.

5) Several major comments from the expert reviewers are appended to amplify these primary concerns.

These are addressed below.

A) Carbenoxelone: unfortunately, we do not have a specific and reversible gap junction blocker, with Cbx being as good as it gets. Nonetheless, the authors should take care in pointing out the caveats (e.g. calcium channel blocking).

We agree that there are caveats to using carbenoxolone and have expanded our mention of these, particularly related to interpreting network behavior, in both the Results (subsection “Electrical synapses between Shox2 INs act as low-pass filters”) the Discussion (subsection “Functional implications of interconnectivity between Shox2 Ins”, first paragraph).

Also, did they try it in any unidirectionally/chemically connected pairs? This might help to tease out non-GJ-related effects.

Carbenoxolone was not tested on unidirectionally/chemically connected Shox2 IN pairs. We agree that this is an experiment we would like to perform but the rate at which unidirectional pairs is found is very low. Although it does not speak to the specificity of the blocker, the other evidence (i.e. hyperpolarization transfer) strongly points to gap junctions being the major, if not exclusive source of coupling. Instead, we tried to get at non-gap junction related effects by performing the opposite, we applied CNQX and APV to bidirectionally connected pairs. Here, no changes were observed in the amplitude of the EPSPs.

Also, on a more minor note, how long was the Cbx applied before effects were seen (subsection “Bidirectional connections may be mediated entirely by gap junctional coupling”)?

Analysis was carried out 30 minutes after washing in carbenoxolone. In some cases, the stimulation protocols were repeated 40 or 50 minutes after the addition of carbenoxolone and there was a further reduction in the EPSPs compared to those after 30 minutes. However, for consistency, all of the analysis included in the manuscript was taken 30 minutes after the drug was applied. This is now explicitly stated.

B) The preparation: it seems that the authors have done experiments both in slices (I assume transverse but they should state this) and dorsal horn removed preps. It is unclear which experiments were which – I think there should be clarity here (throughout the manuscript, but see, for example, the last paragraph of the Introduction – I can't tell what's what).

Thanks for pointing this out. Slices were transverse and this has now been specified in the Materials and methods and the Results. We had compressed the slice and dorsal horn removed data for simplicity, but we agree that the information should be included in the text. Most experiments were performed in slices but the initial P0-5 experiments were performed in the dorsal horn removed preparation. Data from these dorsal horn removed experiments was included in the analysis of incidence, amplitude, and latency. Pharmacology, coupling coefficients, and >P5 experiments were exclusively performed in slices. Bidirectionally coupled pairs were found in both preparations – 46 of 134 pairs in slices and 8 of 84 pairs in dorsal horn removed. We think that the increased incidence rate in slices is due to visibility and our ability to better trace the processes, rather than what remains intact or the rostral-caudal/dorsal-ventral position of one cell relative to the other. It may also be due to the proximity of the neurons in slices compared to the neurons sampled in the dorsal horn removed preparation (see last response to this point). Unidirectional pairs were (perhaps surprisingly) found at relatively similar incidence rates in the two preparations – 2 of 134 pairs (1.5%) in slices and 2 in 84 pairs (2.4%) in dorsal horn removed. These details have been added to the Results section.

Also, how deep were the neurons in the latter preparation?

And do we know anything about the path that their axons take? Subsection “Unidirectional connections between spinal Shox2 INs are sparse”, end of first paragraph – they expect the connections to be preserved but what's the evidence?

The neurons targeted are typically not in the first cell layer at the surface in the dorsal horn removed preparation but they are not far from it, likely 25-150μm from the surface. The depth relative to the dorsal or ventral surface of the intact cord varies since the dorsal horn is removed by hand. In other experiments where this preparation was sectioned transversely after the experiment, the part cut away varied but was rarely far off (dorsal-ventrally) from the level of the central canal.

Shox2 neuronal processes remain local and are primarily oriented medial-laterally (Dougherty et al., 2013, see Figure 1K). Therefore, we expect local connections to be largely preserved in both 300-350 µm slices and the dorsal horn removed preparation. However, we agree that any slicing procedure has the potential to disrupt axons and/or dendrites, which would particularly compromise chemical synapses.

Also, were the distances in connectivity different in the different preps? It sounds like the n's aren't high enough to know, but this should all be clear.

Neurons in electrically connected pairs in slices were closer together (40 +/- 19 μm, n=37) than in dorsal horn removed preparations (56 +/- 20 μm, n=8). Given that neurons in non-connected pairs were not significantly different from connected ones in both slices (49 +/- 14 μm, n=15) and dorsal horn removed (62 +/- 31 μm, n=7) and that the dual patching began in dorsal horn removed, this is likely due to the experimenter becoming more skilled and bolder in patching cells closer together with time (the closer the neurons are together, the more likely one is to disrupt the first seal while going for the second). Although the n’s are too small to say much about unidirectional pairs, we can report the distances for all four cases (25 μm and 48 μm in slices, 32 μm and 65 μm in dorsal horn removed). These values in the different preparations have been added to the text and the graph in Figure 3 has been replaced.

C) Chemical transmission: on the above note, of course it is difficult to prove something doesn't exist. I could certainly imagine that these neurons are connected with one another over longer distances, which could be evident in the dorsal horn removed prep, or in horizontal slices. How is there inter-segmental coordination of rhythm, for example? So I think some rewording acknowledging this – i.e. local connectivity only was tested – would be helpful.

We agree that Shox2 INs are likely connected beyond the very local radii tested. As Shox2 IN processes mainly remain within segment, we do not think these neurons are involved in intrasegmental coordination but we are not testing this and we agree that there must be neurons with such connections. Our bias in testing very local connections is now stated in both the Results (subsection “Unidirectional connections between spinal Shox2 INs are sparse”, first paragraph) and the Discussion (subsection “Shox2 INs are interconnected by electrical synapses”, first paragraph).

D) It is not clear to me that the chemical transmission is monosynaptic. (On that note what temperature were the preps?) How can the authors be sure these aren't disynaptic, thus explaining the low incidence in slice as well as the failures? If they are disynaptic, a whole section of the Discussion can be reduced.

As mentioned above, we cannot say definitively that the chemical transmission is monosynaptic, rather than disynaptic, and have reworded this in both the Results and Discussion. The experiments were done at room temperature, which may also contribute to the high synaptic failure rate. This has been clarified in the Materials and methods. The section the reviewer is referring to was condensed in the reworking of the Discussion.

(Related: subsection “Unidirectional connections between spinal Shox2 INs are sparse”, third paragraph – why is the EPSP so much faster than the EPSC?)

The EPSP latency is shorter because it is measured as the start of the subthreshold depolarization in the postsynaptic neuron but the EPSC latency was measured as the peak of the inward current measured in the postsynaptic neuron. We have changed the wording to make this clear.

E) The authors in several locations discuss examples of coupling between like neurons. But this is not always the case in nervous systems, and in fact 100s of millions of years ago, evolution led to coupling of heterogeneous populations of neurons – which has some significant advantages. This has been seen in many invertebrate systems (see crustacean STG work from Marder lab (Eisen and Marder, 1982), for example), and in the mouse spinal cord (see Wilson, Cowan and Brownstone, 2007).

We agree and initially restricted our statements to rodent spinal cord; however, we did omit Wilson et al., 2007. Thanks for pointing that out. We have reformulated these sections to include that reference and the more recent one from the Zhang and Brownstone groups (Chopek et al., 2018) which also contradicts our original statement.

F) Statistics: I have only one issue with their reporting. I will say (as I always do) that the SEM should be eliminated from neuroscience. It is completely meaningless. We are interested in biological variability, which is shown by the standard deviation. We are not particularly interested in mean values, which are what the SEM reflects. It's great that the raw data are shown in places, but it should always be shown, and I would suggest showing either the s.d. (weaker) or using box-whisker plots on top of the raw data (stronger).

The point is well taken and now all the data is presented as mean ± sd. Additionally, all data points are shown in bar graphs with SD bars (to be consistent with values reported in the text). Raw data sets used in the graphs are appended as requested by *eLife*’s transparent data policy.

And in the first paragraph of the subsection “Shox2 electrical coupling between Shox2 INs declines in incidence and strength with age”: there is no tendency: the statistical test showed that the null hypothesis – that they're the same – cannot be rejected. That's why we use statistics.

The reviewer is correct and we have removed this statement.

G) Conceptual model: It would be nice to understand in the Discussion what the authors are proposing. There are a lot of vague sentences like "intriguing functional consequences." I would suggest they explicitly present a conceptual model. (A computational model would be nice, but that's for the next paper, I would imagine!)

We have made every effort to remove the vague sentences in the revised Discussion. We appreciate the comment and have added our conceptual model as the last paragraph of the revised Discussion. (Stay tuned!)
